# Cerebral Venous Sinus Thrombosis in a Patient with Ulcerative Colitis Flare

**DOI:** 10.1155/2018/5798983

**Published:** 2018-01-21

**Authors:** L. M. Conners, R. Ahad, P. H. Janda, Z. Mudasir

**Affiliations:** ^1^Department of Neurology, Valley Hospital Medical Center, Las Vegas, NV, USA; ^2^Pediatric Neurology, University of Las Vegas School of Medicine, Las Vegas, NV, USA

## Abstract

Inflammatory bowel disease is characterized by a chronic inflammatory state and is therefore associated with abnormalities in coagulation and a hypercoagulable state. Cerebral venous sinus thrombosis is a rare complication of inflammatory bowel disease yet contributes significant morbidity and mortality to those affected. Early diagnosis is critical, as a delay in diagnosis portends a worse prognosis. This paper seeks to highlight the increased risk of venous sinus thrombosis in patients with inflammatory bowel disease. We start by discussing the case of a seventeen-year-old female who presented with ulcerative colitis flare and developed new-onset seizures, found to be caused by a large venous sinus thrombosis.

## 1. Introduction

Inflammatory bowel disease has been shown to be associated with abnormalities in coagulation and a hypercoagulable state [1–3]. The relationship between inflammatory bowel disease and thromboembolism was first described in 1936 by Bargen et al. [4] Since then, several studies have supported the association [5]. The most common sites of these thromboses are in the lower extremity and pulmonary venous systems [4]; however, cerebral venous sinus thrombosis is an accepted rare complication of inflammatory bowel disease (IBD) [2]. Because symptoms of cerebral venous sinus thrombosis (CVT) are oftentimes vague and commonplace, clinicians must heed this relationship in the back of their minds to prevent a delayed diagnosis and possible tragic outcome.

## 2. Case Report

A seventeen-year-old African-American female with mood disorder, infrequent migraine without aura, GERD, and ulcerative colitis presented to the emergency department with four weeks of abdominal pain, hematochezia, and an unintentional 28-pound weight loss over those four weeks. She had been poorly compliant with her medications and follow-up. MRI of the abdomen was notable for diffuse mucosal enhancement of the colon, which contained numerous air fluid levels, consistent with ulcerative colitis exacerbation. Colonoscopy showed multiple ulcerations of the transverse and distal colon. She was hospitalized for three weeks and failed medical management with two rounds of infliximab. Additionally, she required blood transfusions due to intractable bloody diarrhea. Ultimately, the decision was made to proceed with total colectomy and ileostomy, which she tolerated without incident.

**Figure 1:**
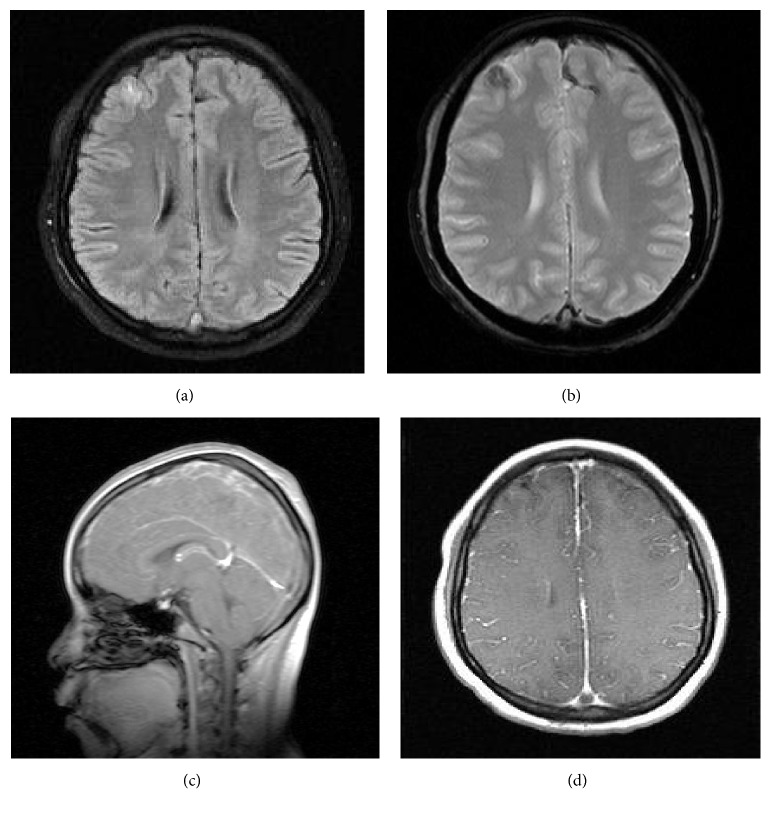
Initial MRI brain of our patient with and without contrast showed right frontal hyperintensity on FLAIR (a) with corresponding area on gradient echo (b), suggestive of a small intraparenchymal hemorrhage. GRE also showed decreased signal of two frontal cortical veins (b). Sagittal T1 imaging (c) revealed heterogeneous signal of the superior sagittal sinus. Postcontrast images were remarkable for a filling defect with direct visualization of the thrombus (d) in the superior sagittal sinus.

**Figure 2:**
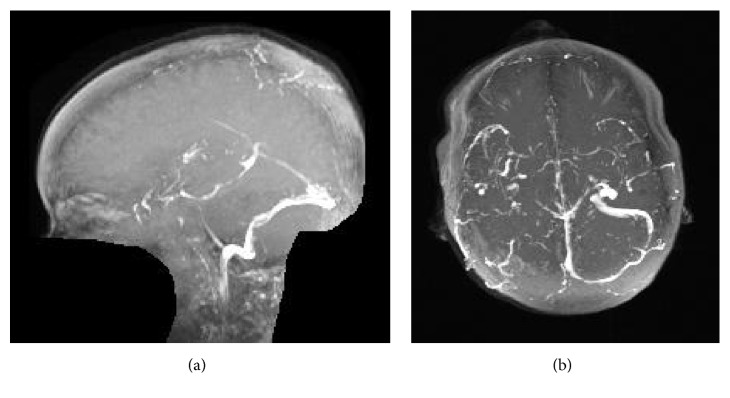
MRV of the head without contrast revealed a lack of flow in the superior sagittal sinus (a), as well as right transverse and sigmoid sinuses (b).

Notably, on her fourth day of hospitalization, she was found down in her bathroom. She was disoriented and complained of a headache. Initial head CT without contrast noted a small subdural hematoma overlying the high bilateral frontal lobes and along the falx. She was transferred to the pediatric ICU, where she subsequently suffered three witnessed tonic-clonic seizures over a twenty-four-hour period. She was treated acutely with lorazepam and loaded on both Keppra and fosphenytoin, and her seizures stopped. The following morning, her repeat CT of the head noted hyperdensity of the superior sagittal, right transverse, and right sigmoid sinuses, raising the suspicion of a venous sinus thrombosis. MRI, MRA, and MRV were therefore obtained. MRA was unremarkable. Her MRI and MRV are shown below in Figures 1 and 2, respectively.

She was started on therapeutic Lovenox with goal Factor Xa level 0.6 since she was anemic from the ulcerative colitis flare. A hypercoagulable panel was negative for protein C deficiency, protein S deficiency, Factor V Leiden, antithrombin deficiency, factor II mutation, and antiphospholipid antibody panel. She was not taking medications prior to hospitalization. She denied a smoking history. Her only known risk factor was ulcerative colitis.

The patient was seen by pediatric neurology while being in the PICU, and her EEG was notable for bifrontal sharps with occasional right temporal sharp complexes. Since her seizures stopped within twenty-four hours, fosphenytoin was discontinued after its load. She was continued on Keppra. Repeat imaging one week later showed significantly improved flow in all three involved sinuses and resolution of the right frontal intraparenchymal and bifrontal subdural hemorrhages.

**Figure 3:**
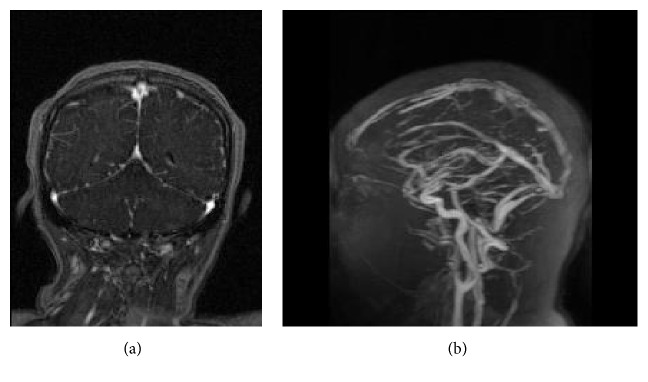
Follow-up MRV of the head 5 months after diagnosis noted minimal residual thrombus in the superior sagittal sinus (a), with resolution of the thrombus in the right sigmoid, and transverse sinuses (b).

She was hospitalized for nearly one month for management of the ulcerative colitis flare, which ultimately required total colectomy and ileostomy. At follow-up, she was doing well. She was continued on Keppra and Lovenox. At that time repeat MRV was notable only for a small residual thrombus in the superior sagittal sinus, as seen in Figure 3.

## 3. Discussion

Cerebral venous sinus thrombosis is an uncommon disease, with incidence between 0.22 and 1.32 patients per 100,000 annually [7, 8]. The prevalence is higher in females than males (approx. 2.5 : 1) [8] (except in children or older adults [9, 10]) and significantly higher in pregnant and postpartum women, 11.6 per 100,000 deliveries [11]. Between 1998 and 2001, Ferro et al. followed 624 adult patients with CVT and noted predisposing factors (Table 1) [6]. Over half had been taking oral contraceptives [6]. Twenty-one percent coincided with pregnancy or the postpartum period [6]. One-third had a hypercoagulable blood disease [6]. 1.6 percent had inflammatory bowel disease [6]. Other risk factors for venous sinus thrombosis include smoking, malignancy, dehydration, substance abuse, infection, and head trauma. Multiple risk factors were found in almost half of the patients [6]. Nearly thirteen percent of patients did not have any clear risk factors [6].

The risk of venous thromboembolism in the lower extremity or pulmonary system for patients with IBD is threefold that of the general population, even after correction for known prothrombotic factors [4]. Cerebral, portal, retinal, and mesenteric veins may occasionally be affected as well [4]. Patients with IBD also suffer thrombotic events at a younger age [4]. The mechanisms of thrombosis in IBD are complex and incompletely understood [4]. Giannotta et al. sought to uncover the mechanism responsible and, instead, found over a dozen differences in the serum of IBD patients, each of which may independently predispose to venous thromboembolism (VTE) [4].  Table 2 summarizes some of those differences.

The presentation of CVT can be highly variable [12]; however, it usually manifests in one of three patterns, symptoms of intracranial hypertension [13], focal neurological symptoms [12], or encephalopathy [12]. Notably, headache is the most common symptom reported in venous sinus thrombosis, with 89% of patients reporting it [10]. The headache is usually gradual [14] and localized, although it often does not lateralize [15–17]. However, when the headache presents as a manifestation of increased intracranial pressure, it is described as diffuse [6]. Monoparesis or hemiparesis is described in 37% of cases [10]. Thirty-nine percent of patient with CVT have seizure upon presentation, and another six percent have seizure within the next few weeks [10]. Seizures are especially common with supratentorial parenchymal brain lesions and sagittal sinus or cortical vein thrombosis [10].

**Figure 4:**
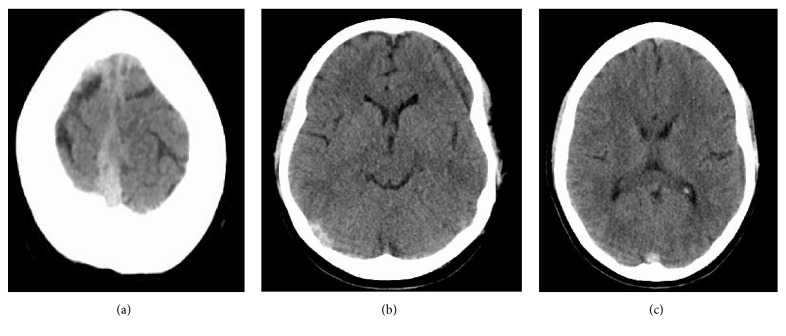
Initial head CT without contrast in our patient described above noted a falcine with bifrontal subdural hematoma at the vertex (a). Note the hyperdense right transverse sinus (b) and “filled delta sign” (c).

Head CT without contrast is the initial imaging modality for patients with acute neurological symptoms [6]. Patients with venous sinus thrombosis will have an abnormal head CT without contrast about 30% of the time [6]. Classically, it may show hyperdensity of a cortical vein or dural venous sinus (Figures 4(b) and 4(c)) [6]. If the thrombus lies in the superior sagittal sinus, one might see the “filled delta sign” (Figure 4(c)) on noncontrasted CT, which appears as a hyperdense triangle at the superior sagittal sinus on axial view [6]. On contrast-enhanced CT, one might see the well-known “empty delta sign,” which would instead show a central hypodensity (due to slow or absent flow) surrounded by contrast enhancement of the sinus [6]. CT may also show edema or infarct, especially abutting a venous sinus or crossing arterial boundaries and may be accompanied by hemorrhage [6]. Approximately 30% of patients with CVT present with intracranial hemorrhage [6]. A prodromal headache, bilateral parenchymal abnormalities, or a hypercoagulable state should also prompt suspicion [6].

**Figure 5:**
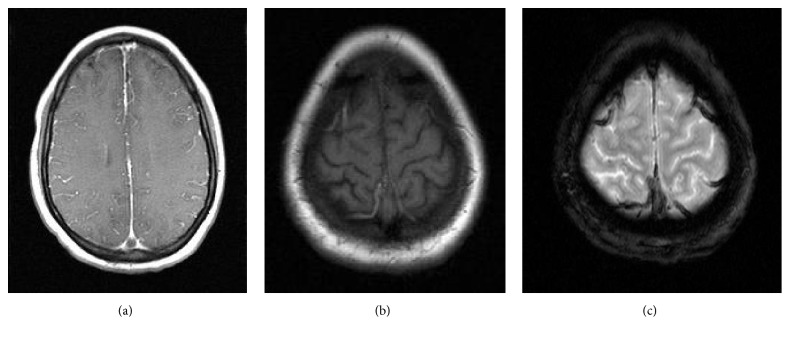
MRI brain with and without contrast showed the MRI equivalent of the “empty delta sign” (a) on postcontrast T1 images. Note the hyperintense cortical veins on T1 (b), which correspond with hemosiderin deposits on GRE (c).

In general, MRI is more sensitive than CT for venous sinus thrombosis [6]. Definitive diagnosis by MRI is made by direct visualization of the thrombus within the venous sinus (Figure 5(a)) [6]. In the first week, the thrombus frequently appears as isointense to parenchyma on T1 and hypointense on T2 [6]. After one week, the thrombus contains methemoglobin and will be hyperintense on T1 and T2, producing hyperintense venous sinuses and/or veins (Figure 5(b)) [6]. Absence of flow void with alteration of signal intensity in the dural sinuses and/or veins should prompt high suspicion [6]. Contrasted MRI can be especially helpful as it can provide direct visualization of the thrombus when it shows a central isointense lesion in a venous sinus with surrounding enhancement (Figure 5(a)) and is the MRI equivalent of the “empty delta sign” on CT [6]. DWI may show infarct, and GRE may show hemorrhage or thrombosed veins (Figure 5(c)) [6].

**Figure 6:**
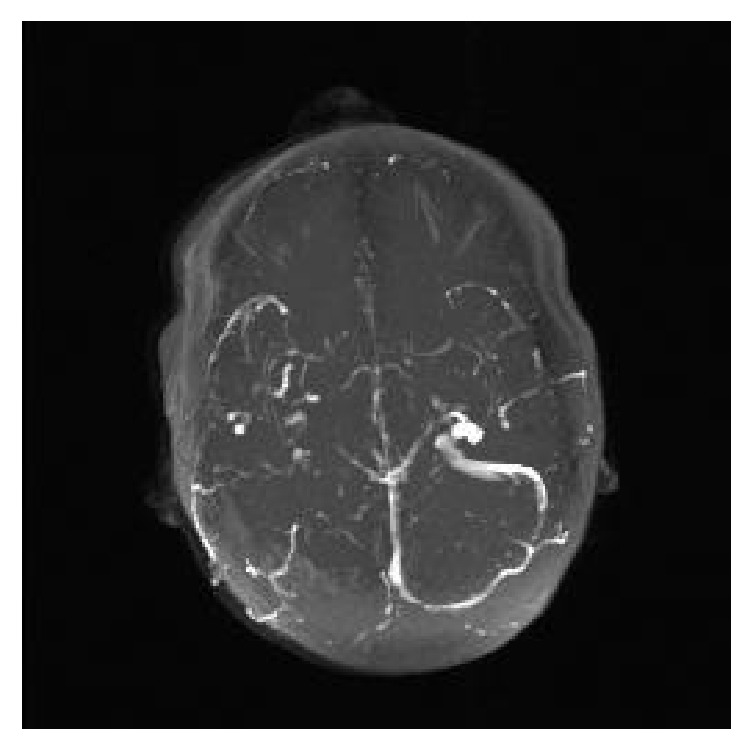
MRV demonstrates absence of flow in the superior sagittal, right transverse, and right sigmoid sinuses in our patient.

MRV is commonly used to aid in the diagnosis in CVT, especially in those for whom radiation or iodine contrast are contraindicated [6]. The most commonly used MRV techniques are time-of-flight and contrast-enhanced [6]. Thrombosis is suggested by lack of flow in the respected venous sinuses [6], as seen in Figure 6. Note that results may be confounded by anomalous venous anatomy, so diagnosis should be made in conjunction with other imaging modalities such as MRI (ideally contrasted) to provide direct visualization of the thrombus [6].

**Figure 7:**
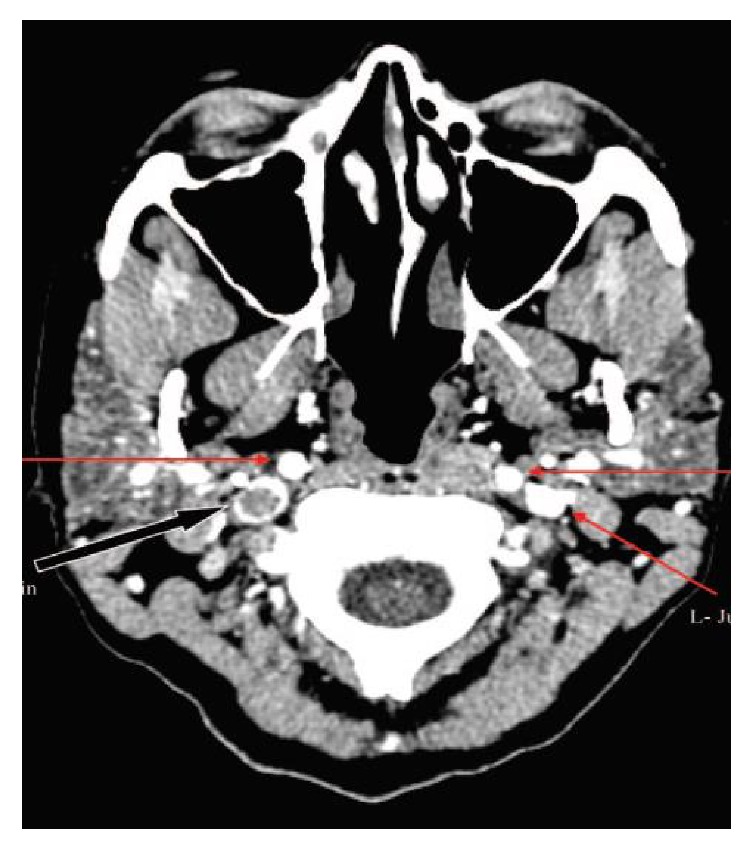
Computed tomographic venogram shows direct visualization of thrombus within the right internal jugular vein in another patient, marked by black arrow [6]. Red arrows mark normal flow voids. Reprinted from [6].

CT venography (CTV) can provide a rapid and reliable diagnosis of CVT [6], as in Figure 7. CTV is at least equivalent to MRV but is limited in a few regards [6]. It may be compounded by bone artifact if the thrombus is adjacent to bone [6]. Pregnant patients represent a large percentage of venous sinus thrombosis patients and should not undergo radiation unless the benefits outweigh risks. CTV is also limited by those who cannot tolerate its contrast due to allergy or poor renal function.

Anomalous venous anatomy, sinus hypoplasia, asymmetrical sinus drainage, and normal sinus filling defects due to prominent arachnoid granulations or intrasinus septa may suggest thrombosis, yet the definitive diagnosis of venous sinus thrombosis rests on direct visualization of the thrombus [6]. In the few patients for whom the above techniques fail to provide a confident diagnosis, cerebral angiography and direct cerebral venography can be used [6]. On cerebral angiography, findings would include the nonvisualization of one or more sinuses, venous congestion with dilated cortical, scalp, or facial veins, enlargement of typically diminutive veins from collateral drainage, or reversal of venous flow [6]. The venous phase of cerebral angiography would show a filling defect in the thrombosed cerebral vein or sinus [6]. Direct cerebral venography by injecting contrast directly into the sinuses or cerebral veins via the internal jugular artery is usually done only in the setting of endovascular therapeutic procedures [6]. One would see a filling defect or complete nonfilling [6].

## 4. Conclusion

Evidence-based medicine supports IBD to be an independent risk factor for venous sinus thrombosis, as numerous studies have demonstrated a correlation [3]. The mechanism for this is multifactorial and incompletely understood [4]. Most healthcare professionals are not aware of this correlation and may not know how to quickly and confidently identify venous sinus thrombosis on various imaging modalities. This paper seeks to highlight the relationship, as a delay in diagnosis of CVT portends a worse prognosis in these young, at-risk patients.

## Figures and Tables

**Table 1:** Risk dactors for CVT [6].

Condition	Prevalence, %^*∗*^	Consistency^1†^	Strength of association^2†^ OR (95% CI)	Biological plausability^3†^	Temporality^4†^	Biological gradient^5†^
Prothrombotic conditions	34.1					
Antithrombin III deficiency		Yes	NA	Yes	Yes	Yes^*ǂ*^
Protein C deficiency		Yes	11.1 (1.9–66.0)	Yes	Yes	Yes^*ǂ*^
Protein S deficiency		Yes	12.5 (1.5–107.3)	Yes	Yes	Yes^*ǂ*^
Antiphospholipid and		Yes	8.8 (1.3–57.4)^*∗*^	Yes	Yes	Yes^*ǂ*^
anticardiolipin antibodies	5.9	Yes		Yes	Yes	Yes^*ǂ*^
Resistance to activated protein C and		Yes	3.4 (2.3–5.1)	Yes	Yes	Yes^*ǂ*^
and factor V Leiden						
Mutation G20210A of Factor II		Yes	9.3 (5.9–14.7)	Yes	Yes	Yes^*ǂ*^
Hyperhomocysteinemia		Yes	4.6 (1.6–12.0)	Yes	Yes	Yes^*ǂ*^
Pregnancy and puerperium	21	Yes	NA	Yes	Yes	NA
Oral Contraceptives	54.3	Yes	5.6 (4.0–7.9)	Yes	Yes	Yes
Drugs						
Androgen, danazol, lithium, vitamin A,	7.5		NA	Yes	Yes	NA
IV immunoglobulin, ecstasy						
Cancer related	7.4	Yes	NA	Yes	Yes	NA
Local compression						
Hypercoagulable						
Antineoplastic drugs (tamoxifen, L-asparaginase)						
Infection	12.3		NA	Yes	Yes	NA
Parameningeal infections (ear,		Yes				
sinus, mouth, face, and neck)						
Mechanical precipitants	4.5	Yes	NA	Yes	Yes	NA
Complication of epidural blood patch						
Spontaneous intracranial hypotension						
Lumbar puncture						
Other hematologic disorders	12	Yes	NA	Yes	Yes	NA
Paroxysmal nocturnal hemoglobinuria						
Iron deficiency anemia		Yes		Yes	Yes	NA
Nephrotic syndrome	0.6					
Polycythemia, thrombocytopenia	2.8					
Systemic diseases	7.2	Yes	NA	Yes	Yes	NA
Systemic lupus erythematous	1					
Baçet disease	1					
Inflammatory bowel disease	1.6					
Thyroid disease	1.7					
Sarcoidosis	0.2					
Other	1.7					
None Identified	12.5		NA	NA	NA	NA

CVT: cerebral venous thrombosis; OR: odds ratio; CI: confidence interval; NA: nonapplicable/nonavailable; IV: intravenous. ^*∗*^Prevalence as per Ferro et al. Percentages for CVT associated with oral contraceptives or pregnancy/puerperium are reported among 381 women ≤ 50 years of age. ^†^Cause-and-effect relationship determined as follows: (1) consistency of association: has the association been repeatedly observed by different investigators (yes/no)? (2) Strength of association: how strong is the effect (relative risk or OR)? (3) Biological plausibility: does the association make sense, and can it be explained pathophysiologically (yes/no)? (4) Temporality: does exposure precede adverse outcome (yes/no)? (5) Biological gradient: does a dose-response relationship exist (yes/no)? Evidence of a strong and consistent association, evidence of biological plausibility, a notable risk of recurrent events, and detection of a biological gradient are suggestive of causation rather than association by chance alone. Modified from Grimes and Schulz. Copyright ©2002 Elsevier. ^*ǂ*^ Evidence for the biologic gradient is not specific for CVT but for VTE.

**Table 2:** Abnormalities in coagulation, anticoagulation, and fibrinolytic system in IBD patients [4].

Coagulation factors	Fibrinolytic factors	Plasma coagulation inhibitors
↑ fibrinogen	↓ tPA	↓ AT III
↑ prothrombin	↑ PAI-1	↓ TFPI
↑ factors: Va, VIIa, VIIIa, Xa, XIa, XIIa	↑ TAFI	Conflicting data about PS and PC
↑ prothrombin factors 1 + 2		
↑ thrombin-antithrombin III complex (TAT)		
↑ fibrinopeptides A and B		
↑ microparticles		
↓ factor XIII		
